# Fundamental Aspects of Lipid-Based Excipients in Lipid-Based Product Development

**DOI:** 10.3390/pharmaceutics14040831

**Published:** 2022-04-11

**Authors:** Deepa Nakmode, Valamla Bhavana, Pradip Thakor, Jitender Madan, Pankaj Kumar Singh, Shashi Bala Singh, Jessica M. Rosenholm, Kuldeep K. Bansal, Neelesh Kumar Mehra

**Affiliations:** 1 Pharmaceutical Nanotechnology Research Laboratory, Department of Pharmaceutics, National Institute of Pharmaceutical Education and Research, Hyderabad 500037, India; dipanakmode@gmail.com (D.N.); 23bhavana11@gmail.com (V.B.); thakor.pradip291@gmail.com (P.T.); jitenderpharmacy@gmail.com (J.M.); pankajksingh3@gmail.com (P.K.S.); 2 Department of Pharmacology, National Institute of Pharmaceutical Education and Research, Hyderabad 500037, India; drshashisingh@gmail.com; 3 Pharmaceutical Sciences Laboratory, Faculty of Science and Engineering, Åbo Akademi University, 20520 Turku, Finland; jessica.rosenholm@abo.fi

**Keywords:** lipid excipients, bioavailability, poorly soluble drugs, lipid-based nanosystems, oral absorption, computational modeling

## Abstract

Poor aqueous solubility of drugs is still a foremost challenge in pharmaceutical product development. The use of lipids in designing formulations provides an opportunity to enhance the aqueous solubility and consequently bioavailability of drugs. Pre-dissolution of drugs in lipids, surfactants, or mixtures of lipid excipients and surfactants eliminate the dissolution/dissolving step, which is likely to be the rate-limiting factor for oral absorption of poorly water-soluble drugs. In this review, we exhaustively summarize the lipids excipients in relation to their classification, absorption mechanisms, and lipid-based product development. Methodologies utilized for the preparation of solid and semi-solid lipid formulations, applications, phase behaviour, and regulatory perspective of lipid excipients are discussed.

## 1. Introduction

Lipid-based drug delivery systems (LBDDS) are formulations consisting of drugs dissolved or suspended in the lipid excipients. LBDDS have been extensively explored for delivering lipophilic drugs, wherein lipids maintain the drug in the solubilized form [1,2]. LBDDS are usually formulated by the dissolution of the drug in the molten mixed lipids and emulsified with water to form a stable lipid system [3]. Solubility screening is most often performed for determining the best lipid excipients for a given drug. Surfactants are used to stabilize the lipid particles by reducing surface free energy or the interfacial energy of the solid particles, thus inhibiting aggregation. Lipid excipients also promote drug partitioning within the skin epidermal lipids and thus accelerate the penetration of drugs through and within human skin [4]. Physicochemical properties and absorption mechanism of the lipids make them different from other classes of carriers leading to substantial cost saving and availability to reach the market.

In this review, we exhaustively cover the lipids excipients useful in formulation development with physicochemical and biochemical behaviour, along with the computational simulations in solubility prediction, phase behaviour, drug absorption and regulatory perspective [5,6]. Lipid excipients are mainly categorized as triglycerides (TG), mixed glycerides and polar oils, water-soluble and insoluble surfactants, co-solvents, and other additives. Ethanol, propylene glycol, glycerol, and polyethylene glycols-400 (PEG-400) are the commonly used co-solvents along with lipid excipients. Surfactants of intermediate hydrophilic-lipophilic balance (HLB) (8–12 lipid excipients, water-insoluble surfactants) that shows adsorption at the oil-water interfaces are available commercially. The lipid-based synthetic surfactant (including non-ionic surfactants), e.g., PEG-40 Hydrogenated castor oil, PEG-60 Hydrogenated castor oil, Polyoxyl 35 hydrogenated castor oil, Polyoxyethylene (20) sorbitan monolaurate and Polyoxyethylene sorbitan monooleate, Glyceryl monooleate, Caprylocaproyl macrogol-8 glycerides, Oleoyl polyoxyl-6 glycerides, and Linoleoyl polyoxyl-6 glycerides are widely used lipid excipients. The water-insoluble surfactant (Propylene glycol monocaprylate, Propylene glycol monolaurate, and Polyglyceryl-3 dioleate) and water-soluble surfactant (Polyoxyl stearate, Polyoxyethylene lauryl ether) has been widely used in the lipid-based formulations [7].

**Scheme 1:**
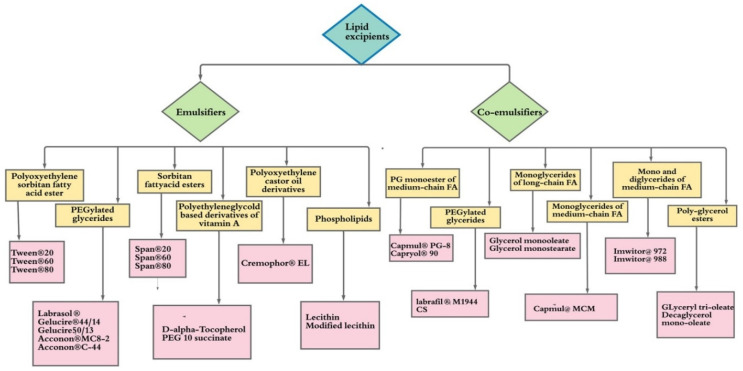
Classification of commercially available lipid excipients.

The nature and type of lipids require further scrutiny since digestible lipids might have an impact on absorption in a different way than that of non-digestible lipids. Lipid vehicles classified based on the chemical structure, polarity of lipids, their characteristics, and the degree of interaction between water and lipids. The class of digestible lipids include phospholipids, fatty acids, cholesterol esters, and several synthetic derivatives [7]. The emulsifier has been classified based on the original nature and shown in Scheme 1. The various available lipid excipients in the lipid-based pharmaceutical product development are summarized in Table 1.

## 2. Lipid Excipients Used in the Pharmaceutical Product Development

The lipid excipients have promising potential in lipid formulation development and their uses are described in this section.

### 2.1. Glyceryl Dibehenate

Glyceryl dibehenate is obtained by esterification of glycerol with behenic acid. It is widely used in the development of lipid-based nanocarriers, e.g., solid lipid nanoparticles (SLNs), nanostructured lipid carriers (NLCs), and nanoparticles.

Abdelbary and Fahmy prepared SLNs by utilizing a modified high shear homogenization process and ultrasonication technique, containing various concentrations levels of glyceryl dibehenate and glyceryl stearate. Glyceryl dibehenate and glyceryl stearate were added as lipid components. It was concluded that the glyceryl dibehenate formed bigger size SLNs along with higher entrapment and significant sustained release of diazepam compared to formulations with glyceryl stearate [21].

Mancini and co-workers explored the application of glyceryl dibehenate in preparation of SLN for topical application. The SLNs were prepared using glyceryl dibehenate as solid lipid and Polyoxyethylene sorbitan monooleate as surfactant using the fusion emulsification method. A liquid (etofenamate) and a solid (ibuprofen) drug molecule is loaded into hydrogel prepared using 2% hydroxypropyl methylcellulose gel by gelation of SLN suspension. The encapsulation of the drug into lipid nanoparticles prevents drug leakage and also protects from external environment. The prepared formulations were found to be of suitable particle size (<250 nm) with encapsulation efficacy 90%. Also, SLN containing hydrogel showed increased drug permeation compared with marketed hydrogel formulation [22].

### 2.2. Dynasan

These pure mono-acid triglycerides are the glycerine esters of saturated, even-numbered, and unbranched single fatty acids like glyceryl trimyristate (Dynasan^®^114), glyceryl tristearate (Dynasan^®^118), and glyceryl tripalmitin (Dynasan^®^116) of plant source. Dynasan^®^ can also be used as a component to regulate the release rate, depending on the concentration [23].

Suvarna and co-workers prepared SLNs using various grades of dyanasan114, 116 and 118. SLNs were prepared by hot homogenization followed by ultra-sonication method. All the six SLNs were prepared using three lipids, each at two different concentrations. Characterization of SLNs was done by measuring particle size, PDI index (poly dispersity index) and zeta potential. By formulating the rosuvastatin calcium solid lipid nanoparticles, an increase in oral bioavailability by 2.2 times compared to a control suspension was observed owing to bypass the effect of first pass metabolism by following lymphatic transport pathway [24].

Gamal and co-workers investigated domperidone-loaded SLN (DOP-SLN) for sustaining the drug release and to improve oral bioavailability. SLNs were prepared using hot homogenization followed by the ultrasonification technique using different solid lipids (glyceryl stearate, stearic acid, hydrogenated palm oil, and Dynasan118^®^). Prepared formulation was characterized physiochemically and evaluated for in vitro drug release and in vivo studies. The oral bioavailability of domperidone SLN prepared using dynasan 118 was around 2.62 times higher compared with conventional tablets. The pharmacokinetic profile in rats (male Wistar Albino rats) revealed that the optimized SLN controlled the absorption of DOP as compared with DOP conventional tablets [25].

### 2.3. Glyceryl Distearate

Glyceryl distearate constitutes of stearic acid and palmitic acid esters. It offers excellent anti-friction properties and is ideal for capsule filling as well for taste masking [26].

Glyceryl distearate is widely used for taste masking mainly by using melt granulation technique and by matrix encapsulation. Forster and co-workers explored twin-screw melt granulation as a strategy for preparing taste-masked ibuprofen granules, using glyceryl distearate as lipid binder. The ibuprofen release from the granules was found to be slower compared with neat API and physical blend. Glyceryl distearate prevent dissolution by forming a physical barrier to dissolution, i.e., matrix encapsulation where drug is encapsulated in a lipid matrix, where a very small amount of the drug remains on the surface which is tolerable on dissolution into the saliva [27].

### 2.4. Glyceryl Caprylate

These mono- and di-glycerides emulsifiers are prepared by the glycerolysis of particular oils, fats, and fractionated vegetable oil fatty acids used in lipid formulation. Kim & co-workers proposes the use of microemulsion formulation for increasing oral bioavailability of Rebamipide. A medium chain glyceride (glyceryl caprylate) was used as lipid phase, ethanol was added as co-surfactant, and polyoxyethylene esters of 12-hydroxystearic acid as surfactant. The microemulsion showed significant enhancement in the dissolution profile and oral bioavailability of Rebamipide without any intestinal toxicity [28].

Bandivadekar and co-workers prepared self-micro emulsified drug delivery system (SMEDDS) with Atorvastatin calcium as a prototypical molecule, which was filled into HPMC capsules. Higher solubilization capacity for Atorvastatin (ATS) calcium was found with glyceryl caprylate amongst other lipids glyceryl monooleate, propylene glycol monocaprylate and propylene glycol monolaurate [29]. These experimentations have proven that the smaller volume oils possess a higher solubilizing potential and self-emulsification ability.

### 2.5. Non-Ionic Surfactant (Lauroyl Polyoxyl-32 Glycerides)

The Lauroyl Polyoxyl-32 glycerides is a family of vehicles derived from mono-, di-, and triglyceride blends of fatty acid PEG esters. Lauroyl polyoxyl-32 glycerides can form microemulsion, i.e., emulsifying to a fine dispersion on contact with aqueous media. It is a non-ionic surfactant dispersible in water. Due to its surface-active property, it promotes enhancement in solubility and wetting ability of API both in vitro and in vivo. For topical formulations, it is used as a coherence agent (thickener) [30].

In a study, Mura and co-workers optimized SLN and NLC formulation of hydrochlorothiazide suitable for paediatric therapy by overcoming the solubility and stability issues. Formulations were prepared using glyceryl distearate and diethylene glycol monoethyl ether as solid and lipid carrier based on solubility results. The impact of several surfactants, in various combinations and amounts, on particle size, homogeneity, and surface-charge of nanoparticles was thoroughly examined. The best formulations were chosen considering drug loading and entrapment efficiency, as well as release behaviour. The use of lauroyl Polyoxyl-32 glycerides as a surfactant instead of PluronicF68 (polyoxyethylene-polyoxypropylene block copolymer) or polyoxyethylene sorbitan monooleate in both the SLN and NLC series resulted in a significant particle size reduction (95–75 nm compared to roughly 600–400 nm), as well as an increase in entrapment efficiency and drug release rate. NLC showed better performance than SLN, with nearly 90% of the drug entrapped (vs. 80%) and more than 90% of the drug released after 300 min (vs. about 65%) [31].

### 2.6. Caprylocaproyl Macrogol-8 Glyceride

The liquid caprylocaproyl macrogol-8 glyceride is a non-ionic oil-in-water surfactant, which is soluble in both oil and water. It has wide application in topical and transdermal formulation for its emulsifying and absorption enhancement property [32,33]. Zhou and co-workers observed an increase in resveratrol oral bioavailability attributable to inhibition of intestinal glucuronidation due to the use of caprylocaproyl macrogol-8 glyceride (labrasol) as a co-emulsifier. Nanoemulsion was prepared using soybean oil as an oily phase, soy lecithin as an emulsifying agent, and caprylocaproyl macrogol-8 glyceride as a co-emulsifier. In vitro absorption study was carried with everted sacs and the results suggested the effect of labrasol on UDP-glucuronosyltransferase inhibition has improved the transfer rate of resveratrol in the everted sacs and the AUC was found to be increased in labrasol nanoemulsion by 2.92 and 5.60-folds in vivo in comparison to negative control nanoemulsion. The superior output of nanoemulsion in the presence of UDP-glucuronosyltransferase inhibitory excipients was attributed to the inhibition of intestinal glucuronidation [32].

### 2.7. Medium Chain Triglycerides

They offer excellent spreadability on the skin and better skin absorption. Caprylic/Capric Triglyceride is used as an emollient, penetration enhancer, drug carrier, solvent, for oral and dermal formulation. The composition of self-emulsified drug delivery system (SEDDS) blend (oil phase) contained CsA, polyoxyethylated castor oil, diethylene glycol monoethyl ether and capric triglyceride. Coating of ethylcellulose and pectin polymers of varying thickness were applied at a low, medium, and high level on SmPill minispheres. Bioavailability obtained from SmPill minispheres was compared with marketed Neoral^®^ PO and Sandimmun^®^ iv of CsA using pig model, after oral administration [34].

In a study, the nanogels that contained methotrexate (MTX)-loaded nanostructured lipid carrier (MTX-NLC) prepared using hot-homogenization. The lipid component constitutes capric triglyceride and glyceryl distearate was used whereas poloxamer 407 was used as a surfactant. Slow and prolonged release of MTX at the end of 48 h (47.32 ± 0.94 percent) compared with methotrexate gel (94.23 ± 0.79 percent) was shown by optimized formulations of MTX-NLC gel. Besides, with the normal recovery of the skin of mice, MTX-NLC gel significantly reduced the Psoriatic area and Severity index (PASI) score [35].

### 2.8. Glyceryl Stearate

The glyceryl stearate with approximately 40%–55% monoester works as co-emulsifier and consistency enhancer in o/w emulsions. Prombutara and co-workers prepared SLNs filled with nisin using high-pressure homogenization to protect from the food environment and to prolong its effect in vivo (slow release). The evidence of antibacterial activity of nisin-loaded SLNs against listeria mono-cytogenes DMST 2871 and lactobacillus plantarum TISTR 850 was observed for up to 20 and 15 days compared to only 1 and 3 days for free nisin, respectively [36].

## 3. Absorption of Lipid Formulations

There are three different major pathways for absorption of lipidic formulations by the body namely lymphatic transport, enterocyte-based drug delivery, reduced pre-systemic absorption. When it comes to orally given lipid nanoparticles (LN), there are two basic digestion and absorption pathways. Lipolysis has been postulated as the primary mechanism of ingested LN in certain investigations. The LN will be digested by lipases and co-lipases after oral delivery, and then converted into mixed micelles for absorption, whereas on the other hand, some studies have discovered that LN can be taken up and delivered as intact nanoparticles across the GI tract [37].

### 3.1. Lymphatic Transport Mechanism

Compounds absorbed via the intestinal lymph shows an increase in bioavailability due to the existence of lipids including both dietary lipids and the lipids presented in formulations. They are transported along with long-chain TGs (triglyceride) lipid centre of intestinal lipoproteins, which are produced in enterocytes after the re-esterification of free fatty acids (FFA) and medium-chain glycerides [38,39]. On the walls of lymphatic capillaries, a single layer of squamous epithelial cells is present, and this thin wall allows tissue fluid (interstitial fluid) or drug to pass through interstitial space to the lymphatic capillaries. Besides, the endothelial lymph vessel structure enables the selective transport of high molecular weight substances such as chylomicrons. The access of chylomicrons through the blood is limited by capillary endothelium. In lymphatic drug transport, the free fatty acid (FFA) chain length and composition of the lymph lipid precursor pool in the enterocyte, play a major role. FFA with a chain length of <12 carbons are normally absorbed through the portal blood, while FFA with a chain length of >12 carbons are re-esterified and transferred through the intestinal lymph. Furthermore, an increasing degree of unsaturation creates the larger size lymph lipoproteins, which leads to the selective enhancement of lymphatic uptake [40,41]. For instance, Hauss and coworkers prepared SEDDS formulations of ontazolast (a potent inhibitor of calcium ionophore), composed of glyceryl monooleate and lauroyl polyoxyl-32 glycerides, and reported that the total amount of ontazolast transported by the lymph over 8 h was almost three times as compared to the control suspension formulation [42].

### 3.2. Enterocyte-Based Transport Mechanisms

**Figure 1:**
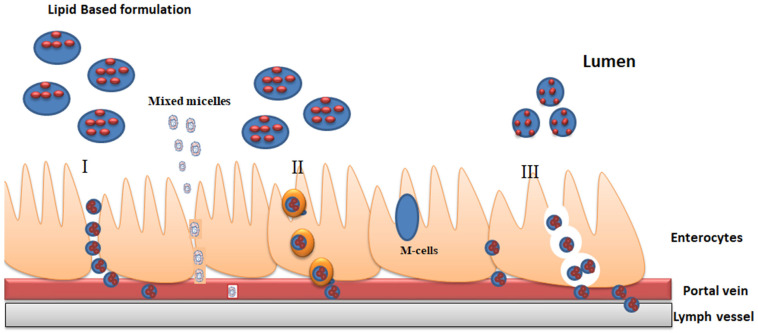
Representation of drug absorption via an enterocyte-based transport mechanism. I—Paracellular transport, II—Receptor-mediated transcellular transport and III—Persorption.

Fatty acids and di-glycerides follow enterocyte-based transport mechanisms such as persorption, receptor-mediated transcellular pathways, and para-cellular pathways via the tight junction [41]. Figure 1 emphasizes the mechanism of enterocyte-based transport, which includes three possibilities [43]. 

Paracellular transport (I)—Drug particles are transported directly through intracellular spaces such as tight junctions. In addition, tight junctions make only roughly 0.1 percent of the intestine’s total absorption surface area.

Receptor-mediated Transcellular transport (II)—It occurs mainly by transcytosis, which is initiated with endocytosis process. Endocytosis is a biological process that involves the transport of extracellular materials into the cell via membrane vesicles. The process begins when nanocarriers binds to the receptors located on the apical membrane, particles are then carried through the cells before being discharged on the basolateral side [37].

Persorption (III)—Extrusion of dead enterocytes from epithelial layers produces holes, which leads to passage of intact particles via holes and this process is known as persorption. Same of the studies have reported GI uptake of numerous big particles by persorption [37].

Numerous lipid-based surfactants used in the lipid-based formulation indirectly inhibit efflux pumps, i.e., p-glycoprotein (p-gp) by affecting the lipid membrane and thereby improving intestinal permeability and oral drug absorption [44]. These inhibitor surfactants include polyoxyl-35 castor oil, polyoxyethylene esters of 12-hydroxystearic acid, polysorbates, PEG esters such as caprylocaproyl polyoxyl-8 glycerides, PEG-6 caprylic and PEG-50 hydrogenated palm glycerides [45,46].

### 3.3. Reduced Pre-Systemic Drug Metabolism Mechanism

Some fatty acids products of lipid-excipients digestion absorbed into the enterocytes are reported to inhibit pre-systemic enzyme activity, which results in a high cellular concentration of the drug [47]. By reducing pre-systemic metabolism, inhibition of CYP enzymes in the intestinal epithelium and liver can significantly influence the bioavailability of drugs that are substrates of these enzymes. The activity of CYP3A4 is mainly inhibit by presence of polyoxyethylene sorbitan monooleate and PEG 400 and thus affected the active drug’s metabolism. Poly-unsaturated-based lipid drug delivery inhibits CYP enzymes in the human body. Therefore, it reduced pre-systemic drug metabolism inside the body and polyunsaturated fatty acid was a potent inhibitor of CYP2C9 and CYP2C19 enzymes [48].

## 4. Methodologies for the Formulation of Solid and Semi-Solid Lipid-Based Formulations

The variety of commercially available lipid excipients has aided formulation designs for LBDDS, which typically function by increasing the oral absorption of the poorly aqueous soluble drugs by formulating emulsion, microemulsion, and vesicular systems. In the last few decades, the conversion of liquid-based formulation to solid dosage forms has been explored, and now it has been thoroughly investigated as the vital subject of pharmaceutical research with in-depth knowledge. Since, for the stabilization of lipid colloidal systems, solidification is the most feasible method, which eliminates the stringent processing requirements related to liquid systems.

### 4.1. Filling into Capsules (Encapsulation)

Hard gelation comprises a lipophilic vehicle, viscosity modifiers for improving the viscosity of liquid vehicles, lipid solubilizer, surfactants, emulsifiers, and absorption enhancers. Lipid vehicle includes arachis oil, cottonseed oil, maize oil, and olive oil, etc. Lipophilic liquid vehicle modifiers include hard fat, emulsifiers, solubilizers, surfactants, and adsorption enhancers. These excipients are compatible with hard gelatin capsules. Softgel fill formulation comprises lipophilic vehicles, which include free fatty acids ( FFA), e.g., fatty acids esters of hydroxyl compounds and oleic acid. The lipid-filled systems consist of drugs dissolved or dispersed in the individual or mixture containing oil, surfactants, and co-solvents. These formulations strategy usually applied for highly lipophilic compounds or potent drugs (with log P > 4) [49,50].

For the soft gel fill formulation, the hydrophilic vehicles include PEGs, methoxy-polyethylene glycols, transcutol, propylene carbonate, tetrahydrofurfuryl alcohol, glycerin, ethanol, N-methyl-2-pyrrolidone, and water. The limit for usage of ethanol, water, and propylene glycol is <10% concentration of the complete formulation fill, since they act as a plasticizer for the capsule shell and can migrate out of the shell [51]. Likewise, the usage of PEG 200 and 300, which are low molecular weight PEG is limited because of their capability of diffusing into the shell besides acts as gelatin plasticizer [52]. A diffusion level of the PEG into the shell from the fill formulation decreases as the molecular weight increases. There is a constraint for the addition of the volatile components (e.g., ethanol) into formulations because they quickly diffuse through shell material or from other filled components that are permitted in the formulation [50].

Heating of reservoir containing solid or semi-solid lipid excipients and retaining formulation in the molten state along with the stirring is requisite before filling. It will help avoid separation of phases and prevent sedimentation if the drug is in the dispersed form. This technique has been explored in the manufacturing of several drugs into soft gelatin capsules (e.g., Bexarotene, calcitriol, dutasteride, lopinavir, nifedipine, nimodipine, and tipranavir) or hard gelatin capsules (e.g., cyclosporin A, ibuprofen, indomethacin, and tolterodine) [53]. The advantages of filling LBDDS in capsules includes straightforward process of formulation, low dose drugs (highly potent) can be filled suitably; and up to 50% *w*/*w* drug loading is possible.

### 4.2. Conversion of Liquid Lipid to Solids

The liquid lipid formulations can be converted to free-flowing powder by adsorption of the lipid formulation on top of solid carrier material. The adsorption method is a simple addition to carriers and involves mixing the liquid preparation with the carrier. The adsorption process is a simple addition onto carriers and includes blending of the liquid formulation with the carrier. The carriers used for solidification are, e.g., calcium silicate, SiO_2_, carbon nanotubes, and neusilin^®^. The selection of the carrier is dependent on its capacity to absorb significant amounts of liquid excipients (to allow high loading of drugs and high exposure to lipids). The resultant free-flowing powders can be filled directly into capsules or instead mixed with suitable excipients for compression into tablet dosage form [53].

Solid carrier acts by direct adsorption of liquid formulation or by the encapsulation of lipid colloids, which are in the dispersed form before drying. Generally, the water-insoluble (highly porous silica-based carriers) and water-soluble (such as polymeric, protein, and polysaccharide) carriers are used as a solid carrier for absorption of a liquid. Solid carriers, such as water-insoluble, silica-based adsorbent highly porous in nature (Flourite RE 7.429 60 20 Neusilin US2 7.429 60 30 Sylysia 320) and water-soluble polysaccharide-based, polymeric, and protein-based carriers are widely used. Good content uniformity and the likelihood of high lipid exposure, for example, up to 70% *w*/*w* of SMEDDS absorption have been recorded to suitable carriers, which is a significant advantage of the adsorption process [54].

SMEDDS thermotropic stability and high drug loading efficiency make them a promising method for low-water solubility drugs, forming small-sized particles. The process is simple and required minimum investment in equipment and more importantly, aids formulation of the tablet, which is globally the most acceptable dosage form [55].

Solidification of lipid colloidal systems is the most acceptable means for the stabilization, with the elimination of strict handling necessities related to liquid formulations. Other advantages of solidification include: (i) Reduced volume of administration if liquid emulsion or suspension consisting of greater than 50% water is converted to the dry powder. It can either be filled into a single capsule or the tablet can be compressed. (ii) Dosage precision is improved, e.g., compared to administration of suspension using a syringe or spoon, pre-encapsulation of the total dose into capsule or tablet offers accurate dosing. (iii) Easy transfer and storage [56,57,58].

### 4.3. Carriers Used for the Solidification of Lipid Formulations

Silica-based carriers are biocompatible, chemically inert, and retain stealth properties [59], and thus evaluated for the formulation of dry emulsion, silica lipid hybrid (SLH) microparticles, and solid SMEDDS [60]. Several amorphous silica or silicate products are available commercially based on varying particle size and porosity (or specific surface area) [61]. The physical adsorption approach is followed with the silica-based carriers for liquid lipids solidification (by simple blending method). Using silica-based carriers, dry emulsion and SLH microparticles are formulated, which are stated to include 30–55% of lipid content and are easily converted into tablet formulation with acceptable tablet friability, hardness, and disintegration. Along with tablet forming aids, the addition of film former and disintegrants is required for ensuring tablets having good matrices and disintegration [62]. Solidification of the lipidic formulation is beneficial using silica-based carrier but the therapeutic activity of parent compound was decreased using these types of solid carriers.

Polysaccharides (carbohydrates)-based carriers have been widely used in food and pharmaceuticals as a sweetening agent, coater, bulking agent, thickness enhancer, binders for tablet, diluents and directly compressing agent. Solid carriers of this class are known for the creation of pro-vesicular formulations essentially owing to cryo-protection property. Nevertheless, the nature of solid carriers (crystalline and hygroscopic) must be taken into consideration while designing the formulation. While processing the polysaccharide-based carriers by spray-drying, lyophilization, and rotary evaporation technique, it is possible that carriers could undergo either partial or complete conversion to the high-energy amorphous form [63].

Polymers soluble in aqueous media shows great solubilization potential for lipophilic compounds. Poloxamers, HPMC, carboxymethylcellulose (CMC), poly-methacrylate, and polyvinyl pyrrolidone (PVP) are some examples of the polymeric carrier. Some polymers act as inhibitors for drug precipitation or promoters of supersaturation. In a study, a low-water-soluble drug Docetaxel (DTX) was loaded in the solid supersaturable SEDDS (DTX-S-sSEDDS) to improve the aqueous solubility and oral bioavailability of poorly soluble drug. This supersaturable SEDDS comprising of the Labrafac, Cremophhore RH 40, Transcutol P and HPMC along with solid carriers. In conclusion, SEDDS shows the improved dissolution and bioavailability of the poorly soluble drug Docetaxel [64,65].

### 4.4. Advanced Formulations Based on Lipids

#### 4.4.1. Self-Emulsifying Drug Delivery Systems

Self-emulsification term is self-explanatory and defines the system’s ability to emulsify the oil phase due to the presence of one or mixture of surfactants. Self-nano emulsifying drug delivery systems (SNEDDS) or SMEDDS are classified based on the droplet size. In a given example, Benznidazole (BZ) loaded SEDDS formulations were prepared for improving the bioavailability of the same. The formulation was prepared using capric triglyceride: propylene glycol monocaprylate: soybean phospholipids (Lipoid^®^ S75): caprylocaproyl polyoxyl-8 glycerides: N-methyl pyrrolidone in (30:15:20:15:20) (% *v*/*v*) ratio where capric triglyceride was used as oil phase, propylene glycol monocaprylate and caprylocaproyl polyoxyl-8 glycerides as a surfactant, and N-methyl pyrrolidone as co-solvent. Approximately 100% of BZ was released into simulated gastric fluid (SGF) media within 6 h in BZ-SEDDS formulation. BZ-SEDDS formulation has shown a better absorption profile compared to suspension of crushed tablets [66].

#### 4.4.2. Vesicular Drug Delivery Systems

**Liposomes** have spherical bilayer structures, which in their arrangement resemble the cell membrane. The lipids mainly used are phospholipids with a hydrophilic head and a hydrophobic tail, which are amphiphilic (fatty acid) [67]. In liposome, amphiphilic phospholipid molecules are arranged in a closed spherical bilayer with fatty acids components facing other molecules. Lipid-soluble drugs are surrounded in “fatty” regions, whereas water-soluble components can be encapsulated in the aqueous core of these globular vesicles [68,69].

**Transferosomes** are modified liposomes, which have the capability of crossing stratum corneum (SC) and penetrating deep into the skin [70]. Transferosomes are the second generation of versatile liposomes with phospholipid and edge activator combinations., e.g., sodium cholate, deoxycholate, and di-potassium glycyrrhizinate. An edge activator typically requires one chain surfactant, which results in the vesicle’s fat bilayer becoming destabilized and improves vesicular fluidity and elasticity. Transferosomes, under the influence of a cross-sectional water gradient, are ultra-deforming or ultra-flexible liposomes that quickly cross the skin. It has been a well-known that, due to the less vesicle material found in the deeper skin layer, liposomes have been restricted to the outer skin layers, i.e., stratum. Hence, different vesicular structures, e.g., transferosomes and ethosomes are formulated where due to their versatile nature such intact vesicles improved penetration to the deeper skin layers [71,72,73].

#### 4.4.3. Solid Lipid Nanoparticles (SLNs)

SLNs resemble solid lipid core, which is stabilized by surfactant in the aqueous phase. Lipid core comprises either single solid lipid or mixed solid lipids at 0.1% to 30% *w*/*w* level disperse in the aqueous phase. The addition of surfactant ensures the stability of the system added at the level commonly from 0.5% to 5% *w*/*w*. Advantages of SLNs include resistance to degradation, sustained drug release, and biocompatibility [74]. Fabrication of the SLNs can be done using a range of lipids, which share some common characters such as high melting point and solidness at ambient and body temperature. Lipids explored for fabrication are mono stearin, stearyl alcohol, cetyl palmitate, glyceryl distearate, glyceryl dibehenate, and glycerol monostearate. Poloxamers 188, and dimethyl dioctadecyl ammonium bromide (DDAB) are the most used surfactants in SLN preparation [75].

#### 4.4.4. Nanostructured Lipid Nanocarriers (NLCs)

They are new generation lipid nanoparticles comprised of a combination of solid lipid and liquid lipids to obtain higher payloads. NLCs improve loading efficiency, stability and prevent the burst release of drugs [76]. Like SLNs, NLCs also offer controlled release for the active moiety along with protection from light, oxidation, and hydrolysis. Besides, NLCs show good biocompatibility and tolerability [77,78].

#### 4.4.5. Mixed Micelles

These types of micelles comprised several molecular species. Mixed micelles resemble a lipid bilayer and show a disc-like structure. Using 1-palmitoyl-2-oleoyl-sn-glycero-3-phosphocholine (POPC) and cholate. Mastrogiacomo and co-workers synthesized mixed micelles filled with lycopene-rich oleoresin (LRO) antioxidants using two slightly different procedures. To generate vesicles of well-defined lamellarity and size, micelle to vesicle transition (MVT) method was employed. The antioxidant capacity of preparations was assessed by calculating the radical scavenging activity of 2,2’-azino-bis(3-ethylbenzothiazoline-6-sulphonate) against the colored radical cation (ABTS). Significant information was obtained on the reliability of different approaches for the evaluation of the antioxidant potential of micelle and liposome preparations and the effective incorporation of LRO antioxidant strength in a bio-deliverable water-dispersed form was shown [79,80,81].

#### 4.4.6. Lipid Drug Conjugates (LDC)

LDC are lipid prodrugs that contain a lipid moiety covalently linked to the drug [82]. Lipid drug conjugate benefits include controlled release of drugs, targeted release of drugs, augmented intestinal permeability, and improved bioavailability. The permeability of drugs incorporated into lipids and stabilized using surfactants is high, since both acts as good drug permeation enhancers in the GIT. Some of the surfactants have the intrinsic property of enhancing intestinal permeability of drug by inhibiting p-gp efflux, which is used for stabilization of the LDC nanoparticles. The LDC nanoparticles show superior loading capacity for nicotine (about 50%) compared to non-LDC SLN (less than 10%), e.g., Berga Care SmartLipids^®^ [83].

## 5. Gaps and Limitations in Development of Lipid-Based Products

While entering into the development, most pharmaceutical companies initially design the pre-formulation and developmental packages for their molecules [84]. This not only helps in assessment of development risks, also provide input into the project plan, including estimated timelines and costs. It provides preliminary direction to the scientific disciplines that will work on the compound during the development phase. Stressed stability testing is a most important investigation, which is used as a warning sign of possible excipient compatibility problems [85].

When it comes to the compounds that are sensitive to oxidation, it is common perceptive that the lipid-based formulations are less optimized because they may contain traces of peroxides that catalysis their degradation. It creates difficulty to acquire required shelf-life of the formulation for supporting the pre-clinical and clinical studies. Generally, two years stability is needed for a commercial product. Although this viewpoint is valid in theory, there are no documented antioxidants that can be added in formulations to prevent this degradation, and it is a general practice in the pharmaceutical industries to check the degradation pattern of lipid based formulation. Nonetheless, reliable testing methods are still crucial, as the relationship between the stability derived from more representative and less stressful parameters like ICH stability conditions, in relation with lipid-based formulations, is mainly unclear [86].

In addition, several parameters are involved in the formulation approach for a new therapeutic molecule in an industrial setting. Solubility analyses is naturally one of the most important investigations, to check if lipid-based excipients have an ability to dissolve the compound. Additionally, stress stability testing results indicate that the drug is generally stable against oxidation, and lipids can be chosen as an excipient. The decision to use a lipid-based formulation would be heavily influenced by the company’s experience as well as stated scientific and production platforms. Thus, defining meaningful formulation composition for the molecule, is not adequately illustrated in public domain. The description of development stories and how to define their composition can provide insight into how to reduce risk perception. For in vitro and in vivo studies, the necessary in vitro characteristics in design of lipid-based formulation for identifying optimized formulation composition as well as relevant quality methods is needed to be explored. More insights are needed into which animal species should be used for study of lipid-based formulations.

## 6. Characterization of Lipid-Based Product Development

The lipid-based product development are the critical and complex product development involve multi-unit operation and critical process parameter (CPP) and critical quality attributes (CQAs) [87] to complete the quality target product profile (QTPP) as per the regulatory approval requirements. The exhaustive characterization is an important aspect of the product development. Various parameter like appearance (colour, odour taste), sterile, pH, particle or droplet size, SPAN value, d_10_, d_50_, d_90_, morphology, lipid contents, encapsulation or drug loading efficiency, assay of drug substances, residual solvents content [88], rheology, viscosity, osmolality, related substances, heavy metals, and elemental profile are crucial in designing. Currently in complex product development, extractable and leachable (E & L) profile of the formulation is a regulatory requirement . Physical stability can be checked by particle size and zeta potential measurement. The SPAN value can be calculated by using [(d_90_ − d_10_)/d_50_] through the particle size measurement equipment and the polydispersity index (PDI), zeta potential can be determine using the dynamic light scattering (DLS) [89] or photon correlation spectroscopy techniques. The surface topography and morphology can be measured by the electron microscopy [90] or atomic force microscopic technique. In lipids-based formulation lipid/excipients ratio and lipid contents is very crucial to determine and can be analyzed by the HPLC, gel permeation chromatography, etc. X-ray powder diffraction (PXRD) for studying polymorphic transformation or crystallinity of the lipid excipients [90]. In vitro studies for evaluation of permeability of oral [91] and topical formulations [90], and in vivo studies are also important characteristics, which need to be established.

The thermal behaviour of the lipid, excipients, polymer and active pharmaceutical ingredients (API) can be determine using the thermal techniques such as Thermal gravimetric analysis (TGA), Differential scanning calorimetry (DSC) [88]. The stability of the lipid-based product to ascertain the shelf-life, degradation behaviour and expiry date with storage condition is required as per the ICH guidelines to mention on the leaflet of the product. Additionally, the various critical evaluation parameters of the container-closure system to pack the product need to be perform as per regulatory requirements.

## 7. Application of Lipid Excipients in Lipid-Based Drug Delivery Systems

The various lipid excipients, which offer numerous applications in delivery of drug have been summarized in Table 2.

### 7.1. Enhancement of Oral Bioavailability

Lipid-based formulations provides an advantage of increased oral bioavailability of poorly aqueous soluble drug using various mechanism. Rifampicin and isoniazid are first-line anti-tuberculosis (anti-TB) medications typically used for tuberculosis therapy. However, isoniazid hastens the hydrolytic degradation in the acidic atmosphere of rifampicin’s poorly absorbed 3-formyl rifamycin SV (3-FRSV) derivative. Further 3-FRSV reversibly reacts with isoniazid and leads to the formation of isonicotinyl hydrazone. The amount of rifampicin remaining for absorption after oral administration is lower, therefore the low oral bioavailability of these anti-TB drugs is reported. The SLNs loaded with anti-TB drugs have enhanced the drug stability in gastrointestinal (GI) environment, besides by-pass first-pass metabolism. Thus, resulting in oral bioavailability improvement by reducing systemic side effects due to decreased dosing frequency [106].

### 7.2. p-gp Efflux Inhibition

p-gp is a trans-membrane protein located at the apical surface of enterocyte, and is responsible for efflux of compounds. The in vivo studies have shown that p-gp is determinant of extent of oral absorption [107]. Surprisingly, the number of excipients, which are used in the formulation of SNEDDS, has a potential to hinder p-gp efflux activity. Furthermore, the entrapment of the drug molecule which is p-gp substrate in the oil droplets would protect the molecule recognition by p-gp transport [100].

The candesartan cilexetil loaded SNEDDS was prepared using peppermint oil, caprylocaproyl polyoxyl-8 glycerides and PEG-40 hydrogenated castor oil to increase the intestinal oral bioavailability by p-gp transporter inhibition. The optimized and successful formulation comprising of peppermint oil 55% *w*/*w*; PEG-40 hydrogenated castor oil 25% *w*/*w* and caprylocaproyl polyoxyl-8 glycerides 20% *w*/*w*, which depicts the best emulsification properties. The optimized formulation shows the prolonged control of blood pressure up to 24 h of hypertensive animal model due to enhanced absorption by opening tight junction and efflux pump inhibitor for more drug absorption. It shows the rapid reduction of systolic blood pressure (136 ± 6.7 to 89 ± 14.6 mm Hg in initial 2 h and depicting the superior activity. Compared to the revolutionary product (ATACAND^®^ tablets), the SNEDDS successfully increased the oral bioavailability of candesartan by 1.69-fold [108].

### 7.3. Enhanced Permeation

Niosomes is a vesicular system commonly used for lipid-based transdermal drug delivery. By changing the structure of the stratum corneum (sc), lipid nanocarriers enhance the drug permeability through the skin [109]. These formulations could prolong the release of drugs thus acts as a drug reservoir, which releases drugs at a steady state. The permeability may be enhanced due to the presence of a larger amount of non-ionic surfactant in niosomes. Improvement in permeability and anti-psoriatic activity was observed for lipophilic celastrol loaded niosomes hydrogel.

Niosomal formulation has been explored in the topical delivery of resveratrol for the pain-related disorder. Thin-film hydration and ether injection methods were employed for niosomes preparation, using surfactant (sorbitan oleate) at three different concentrations. The particle size was observed in range from 214.0 to 331.9 nm Resveratrol was entrapped into niosomal gel, prepared by gelling with carbopol 934. The % entrapment efficiency was reported in the range from 59.2 ± 1.55 to 67.2 ± 1.17 and 46.4 ± 1.21 to 49.6 ± 1.30 for thin film hydration (TFH) and ether injection method (EIM), respectively. The in vitro release studies, ex vivo permeation, and dermokinetic study results showed the potential of the formulation for better cutaneous delivery of resveratrol compared to the plain drug. Resveratrol trapped niosomal gel was found to minimize edema in pharmacodynamics experiments and has extended therapeutic activity relative to the marketed anti-inflammatory gel formulation [80].

### 7.4. Photostability

Being highly photosensitive, Resveratrol readily undergoes degradation upon exposing to light. Encapsulation of resveratrol in lipid could protect the drug against photo-degradation. Pandita and coworkers prepared and extensively characterized resveratrol loaded stearic acid-based SLNs (RLNs) coated with poloxamer 188 by the process of solvent diffusion-solvent evaporation. After 6 h of UV-A irradiation, the drug content in RLNs (86 percent) was considerably higher than the drug solution (36 percent). The findings showed that resveratrol lipid encapsulation effectively protected the drug from photodegradation for a longer period [110].

### 7.5. DNA Delivery

Lipid formulations are promising in oral delivery of both hydrophilic and lipophilic drugs including DNA delivery. Lipoplexes are examples of a complex of DNA with liposomes used in non-viral gene therapy. Lipoplexes (cationic liposomes) are non-viral (synthetic) lipid carriers of DNA. Due to their positive surface charge, they may form complexes (spherical and continuous bilayer structures) with negatively charged DNA [111].

## 8. Administration Routes of Lipid-Based Formulation

### 8.1. Oral Delivery

Particle size has strong influence on the uptake of non-soluble particulate matter through the digestive tract. Microparticles in the gut lumen can release drug, which are then absorbed into the body by persorption. The absorption of NPs, on the other hand, has piqued the interest of researchers working to find effective carriers for oral absorption of drugs that are either poorly absorbed or sensitive to gastrointestinal degradation [112]. Schultz and co-workers explored the supersaturation technique for improve the drug loading in lipid-based formulations. Thus, the silica-lipid hybrids are solid lipid-based formulations developed mainly for improving an oral delivery of poorly soluble drugs. They prepared supersaturated silica-lipid hybrids using poorly water-soluble ibuprofen (IBU), based on solubility in lipids propylene glycol monocaprylate was selected as the lipid, lipid and silica were used in 1:1 ratio for loading ibuprofen. The drug loading of 8–44% *w*/*w* was achieved, which was greater than previously developed ibuprofen silica-lipid hybrids (5.6% *w*/*w*). It was discovered that a new fabrication method for supersaturating SLH (silica–lipid hybrids). The IBU load was higher than previously reported, indicating a considerable advancement in overcoming the IBU loading limitation in solid-state LBFs. When compared to pure ibuprofen, Super-SLH produced higher rates and extents of dissolution, regardless of the drug content. At 60 min, the percentage increase in dissolution extent ranged from 200 to 600 percent. The current study’s findings shows that supersaturation increases drug loading and that 16–25 percent *w*/*w* is the optimum loading level for ibuprofen oral administration while maintaining good dissolving behaviour, which might be applied to other poorly water-soluble medicines [113].

Buparvaquone (BPQ) used in treatment of Visceral Leishmaniasis available in market as I.M. injection, but the orally administered formulations have limited success. Lindsay and co-workers investigated the formulation of buparvaquone SNEDDS for oral delivery. The prepared formulation consisting of buparvaquone, oleoyl macragol-6 glycerides as oily solubilizer, co-surfactant propylene glycol monocaprylate and caprylocaproyl macragol-8 glycerides as surfactant in ratio of 0.01:3:1:5.99. The prepared SNEDDS were obtained with high drug loading and with 6 months stability. The in vitro studies were carried out using flow through cell apparatus and the vitro-lipolysis test revealed that the SNEDDS has increased the solubilization capacity of buparvaquone in GI media. The prepared SNEDDS demonstrated enhancement in oral bioavailability comparative to the aqueous dispersion of drug (Male CD-1 outbred mice) resulting in AUC_0-24_ that are four folds. The addition of caprylocaproyl macragol-8 glycerides as surfactant has proved the ability of surfactant to enable diffusion through mucous layers [114].

Olmesartan is a BCS class II drug with poor aqueous solubility. Pandya and co-workers prepared Olmesartan medoxomil loaded SLNs with the objective of enhancing the oral bioavailability of the drug. SLN were developed utilizing a heat homogenization process. The formulation variables lipid (Glyceryl monostearate) and surfactant were optimized using central composite design (Poloxamer 407: polyethylene sorbitan monooleate). The optimized SLN formulations were of nanometric particle size (121.20 ± 3.5 nm) with spherical in shape with high zeta potential indicating good long-term stability. The controlled release profile of olmesartan loaded SLN was seen for at least 24 h in vitro. The rate and extent of drug diffusion were investigated using dialysis sac, rat stomach, and intestine tissues. The results showed that drug release from SLN was much higher than drug suspension. When compared to commercial formulations, in vivo pharmacokinetic studies using olmesartan loaded SLN exhibited greater C_max_ of 1610 ng/mL, and enhanced relative bioavailability by about 2.3 times. It would seem reasonable that SLNs, due to their small particle size and sustained release, might be easily absorbed from the duodenum to the lymph, allowing the drug to enter the systemic circulation and produce the desired therapeutic effect [115].

### 8.2. Topical Delivery

Topically applied drug-loaded nanoparticles are mainly intended for local effect, to prevent the drug administration systemically, and to lower the amount of dose required to reach therapeutic level at the targeted site, i.e., skin thereby reduces the off-target effects [112]. The stratum corneum (SC), the epidermis’ outermost sublayer and a physical barrier to substances contacting the skin, is a critical issue in cutaneous delivery. For overcoming the absorption constraint caused by SC, suitable carrier systems for topical administration of multiple drugs are required.

Maghrabi and coworkers [116] developed the Miconazole nitrate (MN)-loaded solid lipid nanoparticles for topical delivery to increase the effectiveness against the fungal infection. MN loaded SLNs were prepared by using high shear homogenization technique. The prepared formulations were evaluated for entrapment efficiency (EE), poly dispersity index (PDI), particle size and zeta potential. Finally, the optimized formulation containing gelucire 39/01: GMS(glycerol monostearate) 1:1 ratio as lipid, PEG-40 hydrogenated castor oil (5%) as surfactant and transcutol as co-surfactant (1%) showed particle size range 244.2 ± 27.2 nm to 493.6 ± 35.3 nm, EE up to 95% and SLNs were of spherical shape. Later, formulation was evaluated for in vitro release, ex vivo studies, skin toxicity test and anti-fungal activity. The in vitro release studies showed controlled release profile up to 48 h. The optimized formulation showed higher miconazole nitrate flux in the skin compared to MN solution in ex vivo studies. Furthermore, when compared to a simple MN solution, selected MN-SLN exhibits a larger zone of inhibition against Candida albicans. MN-SLN showed the lowest skin toxicity value. Furthermore, compared to MN-solution on immune-suppressing albino rats with generated candidiasis fungal infection, the selected MN-SLNs significantly reported antifungal efficacy with the least histological improvements [116].

Hamed and co-workers combined the two systems, the microemulsion and the organogel to explore the application of this combined system in the delivery of lipophilic drug Lidocaine. The O/W type microemulsion was prepared by dissolving lidocaine in oil phase and the emulsion was stabilized by using lipid surfactant. The oil droplets were reduced to submicron size to improve the skin penetration. The prepared microemulsion was gelled using glyceryl behenate as organogelling agent. Based on the droplet size and physical stability of microemulsions, optimised formulations of lidocaine microemulsions with oil, water, and surfactant:cosurfactant (polyethylene sorbitan monolaurate:ethanol) at 4:1 and 2:1 *v/v* were chosen. As a control, Lidocaine standard organogel was made without the inclusion of microemulsion. Lidocaine organogels rheological characteristics and release profiles were examined. The viscoelastic characteristics of Lidocaine conventional and microemulsion organogels were shown to be more elastic. When compared to microemulsion organogel containing polyethylene sorbitan monolaurate:ethanol (2:1 *v*/*v*), microemulsion organogel containing polyethylene sorbitan monolaurate:ethanol (4:1 *v*/*v*) displayed lower viscoelastic characteristics and higher rate of release [117].

The encapsulation of the API into the lipid-based nanoparticles with particle size < 100 nm, could be used for enhancement of lymphatic uptake after intradermal administration. To explore this approach, Permana and co-workers developed dissolving microneedles containing antifilariasis drugs (doxycycline, diethylcarbamazine and albendazole) loaded SLNs. The SLN were prepared using glycerol monostearate as lipid matrix and polyethylene sorbitan monooleate as stabilizer. SLN prepared using glycerol monostearate showed high encapsulation efficiency, owing to the formation of hydrogen bonds between two hydrogen bond donor functional groups in glycerol monostearate (hydroxyl groups of glyceryls) and hydrogen bond acceptor functional groups in doxycycline, diethylcarbamazine and albendazole, which could result in a molecular complex Using a central composite design, the formulas were optimized to produce SLNs with diameters of less than 100 nm. In comparison to pure drug substances, drug release from SLNs was sustained for 48 h. The SLNs were then mixed with a polymeric hydrogel and cast to create SLN-loaded microneedles (MN). MNs filled with SLNs exhibited adequate mechanical and insertion properties. Importantly, dermatokinetic experiments indicated that >40% of drugs were retained in the dermis of excised newborn pig skin for up to 24 h after MN administration, showing that the SLNs are highly likely to be absorbed by the lymphatic system. The maximum lymph concentrations of the three drugs in rats reached after intradermal delivery was varied between 4 to 7-fold greater than those recorded after oral administration in in vivo experiments. Dissolving MNs are a type of drug delivery method that can bypass the stratum corneum, the main skin barrier, and are thus regarded as innovative intradermal drug delivery systems [118].

### 8.3. Pulmonary Delivery

Because of the high vascularity and circumvention of the first-pass effect, the pulmonary route provides a non-invasive form of drug delivery as well as a large surface area with quick absorption. Inhalation enables localized delivery for lung-related diseases, enhancing treatment efficacy at the site of action. These lipid-based micro or nanoparticles being used to treat the pulmonary-related diseases. Inhalable microparticles are generally prepared using spray-drying [119,120,121]. Umerska and co-workers prepared lipid nanocapsules-trojan particles in carbohydrates-microparticles using spray-drying technique with particle size 59 ± 3 nm. These LNCs prepared by the polyoxyl-15-hydroxy stearate (MHS), hydrogenated lecithin, and caprylic/capric acid triglycerides (TGs). These microparticles show promising delivery of bioactive using dry powder inhalation dosage forms for pulmonary disease [122].

Messenger RNA (miRNA) is a promising class of nucleic acid therapeutics that can be used to treat a wide range of ailments, including genetic disorders. The creation of a stable and effective mRNA pulmonary delivery system would allow for high therapeutic concentrations to be achieved locally in the lungs, improving efficacy while reducing potential toxicities. Zhang and co-workers used design of experiment for screening the numerous combinations for optimizing lipid nanoparticles formulation, finally to get formulation with high potency before and after aerosolization. Lipid nanoparticles (LNPs) was found to be stable physico-chemically after being stored at 4 °C for at least 14 days, and most formulations had high encapsulation capacity of more than 80%. Formulations were found to have higher intracellular protein expression capabilities in vitro, even after aerosolization, and were tested in vivo on Balb C mice. For the four lead formulations, luciferase protein was shown to be primarily produced in the mouse lung before and after nebulization. This work showed that LNPs have the potential to be used for pulmonary mRNA delivery via aerosolization [123].

Ethambutol hydrochloride (EMB) is an anti-tuberculosis medicine that is frequently used as a preventative measure against the development of undetected resistance to other drugs used to treat the lung disease. The oral form of EMB has some side effects and cellular toxicity, and thus administering EMB directly into the lungs appears to be an appealing and reasonable option for overcoming these issues. Nemati and co-workers investigated the pulmonary administration of EMB-loaded SLNs via a dry powder inhaler (DPI). They used two methods to make EMB-loaded SLNs (i.e., hot homogenization and ultrasonication) using glycerol behenate as solid lipid. Spray drying EMB-loaded SLNs with and without mannitol yielded DPI formulations. Carr’s index and Hausner ratio were used to investigate the flowability of the produced powders. The resulting particles encapsulation efficiency was found to be greater than 98% with a particle size less than 100 nm. Toxicity testing of EMB-loaded SLNs was carried out using the MTT assay, which revealed that the SLNs are biocompatible and non-toxic. Overall, the findings revealed that EMB-loaded SLN DPI has a high potential for direct tuberculosis treatment [124].

### 8.4. Parenteral Delivery

Parenteral administration is a typical method of delivering the active drug substances that have low oral bioavailability and therapeutic index [112]. The lipid-based nanoparticles including SLN, NLC and lipid-drug conjugates are gaining significant attention owing to their stability, large scale-up manufacturing, non-toxic and non-immunogenicity. A few lipid-based products, e.g., Doxil^®^ and AmBisome^®^ are available on the market with the high therapeutic efficacy and safety data [125].

Ziprasidone (ZP) is a novel atypical antipsychotic drug that is beneficial in combating both positive and negative symptoms of schizophrenia. ZP has a low oral bioavailability of 50%, a short biological half-life of 2.5 h, and a repeated dose is required due to substantial first-pass metabolism, making the therapy non-adherent and contributing to patient non-compliance. As a result, Khan and co-workers explored the parenteral ZP-loaded sustained-release phospholipid-based phase-transition system (ZP-LPS). Biocompatible ingredients such as phospholipid E 80 [126], medium chain triglyceride (MCT), and ethanol were mixed to prepare ZP-LPS system. Aqueous titration with a pseudo-ternary phase diagram and dynamic rheological data were used to optimise the process. Gamma scintigraphy following subcutaneous injection confirmed the generation of in vivo depots. Finally, Sprague Dawley rats were used to test the formulation’s effectiveness. Optimized ZP-LPS gelled quickly (2 min), had the largest viscosity change (mPa.s), and released ZP for 1 month. The low-viscosity ZP-LPS system gels rapidly in situ, according to gamma scintigraphy results [127].

Curcumin, which has a wide spectrum of biological and pharmacological properties such as antioxidant, anti-inflammatory, anti-tumor, anti-carcinogenic, anti-bacterial, and anti-HIV activity, has low water solubility and low bioavailability [128]. Saedi and co-workers investigated effect of various lipids on different features of NLCs for delivering curcumin. Coconut oil, fish oil, black seed oil, and linseed oil were used as liquid lipids, whereas cetyl palmitate was used as a solid lipid in all NLCs formulations. All the NLCs were analyzed for particle size, zeta potential, polydispersity index (PDI), drug entrapment percentage, and drug loading capacity. The NLC containing black seed oil had the smallest size, according to the results. Other characteristics, such as PDI, zeta potential, and entrapment efficiency, were consistent across all liquid lipids. In comparison to the other NLCs, the NLC containing black seed oil showed the highest % of drug release and antioxidant activity. According to Higuchi’s drug release kinetics, diffusion was the main mechanism of drug release [129]. When compared to free curcumin, the curcumin loaded NLC of linseed oil demonstrated better cytotoxic efficacy in a cell viability experiment on MCF-7 cells.

### 8.5. Implantable Drug Delivery System

An implantable drug delivery system, which includes SLN-based alendronate in situ gel formulation for local therapeutic activity utilizing a thermogelling system was developed and reported. Alendronate-loaded SLN (ALD-SLN) prepared by high pressure homogenization (HPH) followed by ultrasonication. Ultrasonication and HPH have been using for micronization (particle size) of API. The formulation was prepared using lipid phase glyceryl monostearate (GMO) and polyoxylethylene sorbitan monooleate was used as a surfactant, while Pluronic-127 and Pluronic-68 (PF-68) were used as the polymer. The implantable system could overcome the risk of oesophageal ulceration, which is the drawback of the conventional dosage form. The prepared ALD-SLN-based in situ gel formulation was proved to be an efficient delivery system for osteoporosis treatment [130].

## 9. Regulatory Perspectives

Earlier it was reported that excipients are considered as inert components, which mainly used as a tablet binder, lubricating agent, diluents, coating agent, vehicle, and dyes in the production of the pharmaceutical product. The advancement in pharmaceutical science and technology has eased the accessibility of wide-ranging novel excipients. Specific excipients may show known or unknown interaction among excipients and actives, other inactive ingredients, or a container closure system (CCS). Therefore, currently, it is predictable that not all the excipients are inert, but few may exhibit potential toxicity [131].

In the Code of federal regulations (CFR), a list of substances is published by United States of Food & Drug administration (US-FDA), which is generally recognized as safe (GRAS) prominence. Additionally, it holds the materials lists, which are inactive components for excipients so-called inactive ingredient guide (IIG). This IIG guide also enlisted the maximum concentration tolerable along with a specific route of administration. These GRAS and IIG list provide information to the formulator for development of any new and generic pharmaceutical drug product. As excipients are the essential component of the formulation, no specific review process is followed differing from drug products and the regulatory process is suitable from a scientific perspective [132].

Once the excipient is approved in the drug product for a particular route of administration during new drug development, the inactive ingredient is no newer for the formulator. A less extensive review process is required for next time to use it in the dosage form development. Clinical and non-clinical studies have been required in terms of safety to use of new excipients in product. In this perspective, the guidance document for the industry was published from USFDA for the conduction of non-clinical studies for evaluating novel pharmaceutical excipients’ safety. The guidance also offers the safety evaluation parameters for excipients used in over the counter (OTC) drugs and generic product and explained the type of toxicity data used in the determination of the safety of new excipients. Significantly, this guidance has highlighted the need for performing risk to benefit assessments on the approved excipients in the drug product. There is currently no FDA process or framework available for independently testing of new excipients so far. Instead, excipients used in the drug products or biological formulation, which are in the premarketing approval process are reviewed and accepted as “component” of drug products or the biological formulation in use. Because of their distinct physicochemical properties and possible complex interactions with other ingredients or the physiological environment that may occur in vivo, this is particularly true for lipid excipients. Conversion of oils or lipids into cytotoxic agents is possible due to reduction to nano range in situ (e.g., in SNEDDS), and thus formulators need to investigate in detail the oils used in such a system [133].

International Pharmaceutical Excipients Council (IPEC): The aim of the IPEC guide is to regulate the content of the Certificate of Analysis (COA) and to recommend the organization of excipients. The functions and responsibilities of the excipients manufacturers and distributors were also defined. The first original guidance was published in year 2000, but due to the changing global pharmaceutical market and the regulatory area, a revision of the guide was released in 2013. COA guidance has been available in USP32/NF-27 as the chapter (1080) bulk pharmaceutical excipients-COA. Besides, IPEC released an excipients information package (EIP) that includes information relating to excipients qualification and sourcing in the regular package. Due to the EIP kit, the need for the number of questionnaires and surveys needed to gather information from different sources is reduced. The EIP package was set up in the same way as that of the material safety data sheet (MSDS), which comprises titled segments to contain certain data on subject. The EIP package includes an overview of site quality and a datasheet of product regulatory and supply chain security [134].

## 10. Computational Prediction

### 10.1. Formulation Design and Development

The molecular dynamic (MD) simulations play an important role in the pre-formulation screening of the carriers, polymers, excipients and identification of the drug molecule. MD mimics atoms and molecules physical motion under Newton’s physics laws [135]. Based on the reliable prediction results and correlation with the experimental data make these approach more reliable, process time and cost-effective. All mathematical models depend on the generation of actual data relating to the formulation of interest. This data can include in vitro, iv vivo and clinical data as well as physicochemical data relating to formulation of APIs [136].

To begin with, the carrier’s ability to retain its cargo (drug payload) is the critical aspect for a successful lipid formulation. Pharmaceutical formulation designing by in silico method is becoming an important aspect of modern drug delivery research. It is becoming more popular since it gives researchers a highly pixilated image of their target based on molecular characteristics of both the drug and the carrier [137].

Metwally and Hathout used MD to simulate the loading of 21 API in tripalmitin and polylactide-co-glycolide (PLGA) 50:50-based nanocarriers using a larger dataset that included a lipid carrier. Where ten drug molecules were set for PLGA system and eleven for tripalmitin. They used all-atom MD simulation (in GROMACS software) and docking (ArugsLab and Autodock Vina). The clear correlations were obtained between the docking ΔG data for drug loadings in tripalmitin and PLGA and their respective documented drug loadings. In addition, the authors used model drug curcumin to assess the predictability of the developed model. For tripalmitin SLN and PLGA nanoparticles, the projected percentage bias between actual and projected loading values was 12% and 2.03%, respectively [137].

Haoshi and coworkers in 2021, acquired dataset for SEDDS formulation comprising 4495 composition ratios. Models were constructed with 7 machine algorithms for determination if the oil, surfactant, and co-surfactant can form the SEDDS. In a five-fold cross-validation, random forest has the best accuracy, sensitivity, and specificity in comparison to other machine learning algorithms, with 91.3% accuracy, 92.0% sensitivity, and 90.7% specificity. For further screening of the ratios, the DOE method of central composite design (CCD) was applied (PBPK) [138]. The molecular interaction between drugs and excipients can be studied using MD simulation, which demonstrates the diffusion behaviour in water and role of co-surfactant. Finally, for rational SEDDS formulation design, this study incorporated machine learning, central composite design, molecular modelling, and experimental techniques. The molecular simulation can imitate the physicochemical process at molecular scale. Gradually, MD simulation has become more effective tool to aid in understanding the solubility, dissolution mechanism and target specific delivery [139].

### 10.2. Pharmacokinetics Prediction

New modelling tools that can simulate the interaction of emerging molecular structures with the biological macromolecules are available. The number of conditions is necessary to consider in the designing and development of nanomedicines such as stability of formulation, control drug release rate and targeting ability [140]. The MD models merged with the PBPK (Physiologically based pharmacokinetics) or QSP (quantitative structure property) models for creating effective approaches will characterize not only the disposition and activity of the compound of interest but also the activity and interaction of various excipients existing in the administered formulation with the different biological matrices across the body. Such models integration across several computational tools can give a completed image of the formulation pharmacodynamic (PD) [136].

The PK software such as SimCyp, GastroPlus, and STELLA can be used to analyze the performance of formulations in silico. Shea and co-workers [141], as well as Zheng and co-workers [142] presented examples of current computer guided lipid based formulation (LBF) performance studies. It should be noted that for these simulations to be correct, a certain amount of in vitro data is typically required as input, and that totally in silico approaches are still out of reach [143]. The PBPK method is a valuable tool for evaluating drug formulation PK characteristics.

For orally administered drugs, the prediction of PK profile can be done with PBPK model, which has benefited from the advancement of in vitro experimental methodology [135]. PBPK modeling is type of PK prediction technique which can depict the concentration versus time profile of the drug in the body, which is bases for the correlation of therapeutic or toxic effect of drugs. The PBPK consists of numerous compartments which are likely to be linked by the differential equations, which represents that the body organs are connected to the circulating system. For calculating PK profile, the model parameters include drug physicochemical properties such as lipophilicity, pKa, solubility and molecular weight or physiological properties which include tissue volume, blood flow, transporters, and enzymes, etc. [144]. As a result, PBPK modelling is easy to elucidate and allows modifications according to real conditions.

An integrated PK/PD model is reported in a study of an mRNA therapy, uridine-diphosphate-glucuronosyltransferase (hUGT1A1)-modRNA. In this study, mRNA was administered through lipid nanoparticle (LNP), delivering the UGT1A1 for the treatment of Crigler Najjjar syndrome type 1. Classical compartment model was used for the simulation of PK part, i.e., LNP elimination from the plasma, whereas PD models mimics the LNP endocytosis by liver hepatocytes, mRNA release from endosome, mRNA translation into UGT1A1, and UGT1A1 clearance of unconjugated bilirubin, which is a disease biomarker. For finding the proper dose for first-in-human clinical trials, this model can be used [145]. Additionally, while developing PBPK models, particular nanoformulation properties should be taken into consideration necessitating the use of novel algorithms and modelling strategies.

### 10.3. Phase Behaviour during Dispersion

A MD simulation delivers an unprecedented view of lipid-based formulations structure, possesses extensive possibilities as an in-silico tool for the formulators and can be used to study the phase behaviour of liquid and liquid crystal systems. Among the available application areas, MD has great potential as a method for predicting the microstructure of lipid-based drug formulations [146,147]. MD specifically provides detailed information of molecular interaction within formulations, which could not be studied by X-ray diffraction method or spectroscopic techniques [148,149].

Warren et al. investigated the microstructure of lipid-based formulations or their structure changes in the gastrointestinal tract. The basic lipid formulation system was investigated alone and in the presence of the drug. The composition of the formulation includes acyclovir, danazol, hydrocortisone, ketoprofen, mono- and dilauroyl glyceride at 0–75% water for dynamic behaviour and localization during dispersion was evaluated. GROMACS (Groningen Machine for Chemical Simulation) software was used for performing MD simulations [150,151]. The simulation trajectories were visualized using VMD (visual molecular dynamics). The programs used for the analysis of MD trajectories were part of the GROMACS software package. Improved MD techniques have been developed that include atomic-scale drug dispersion and dynamics model, enhanced MD provides first drug dissolution simulations inside the lipid formulation/water framework. Three separate phase regions in the range of 0–75% *w*/*w* water were revealed from the simulation studies of Type I formulation [152]:

(i) A phase like single reverse micelle comprising included water molecules on the lower concentration of water,
(ii) Single reverse phase of micelle at intermediate and high-water levels and,
(iii) The lamellar glycerides a two-phase system and lose water groups.

Simulation experiments in the formulation or water systems of poorly water-soluble drugs such as hydrocortisone, ketoprofen, danazol, acyclovir, and progesterone have shown that drug localization is driven by confined hydrophobic/hydrophilic properties of the drugs only at all levels of water concentration. Drug localization is a means of establishing localized hydrophobic interactions with water, formulations, or other drugs within the system. Hydrophilic drugs, e.g., acyclovir has hydrophobic faces which allows interactions with the lipid formulation. For acyclovir, it was observed that the polar portion of the drug possesses the ability to interact with water or polar lipid atoms, whereas the less hydrophilic region, i.e., aromatic ring surfaces can be protected from the aqueous environment by interacting with lipids. Analogous behaviour was detected in the case of other polar drugs as well, e.g., ketoprofen and hydrocortisone. The drugs with fewer bonding groups of hydrogen display deeper binding in the alkane tail of the lipid section. Progesterone containing two hydrogen bond acceptor atoms exists deep inside the lipid portion. While the more hydrophilic danazol molecule consisting of 1 donor of hydrogen bond and 3 acceptors of a hydrogen bond is presented inside the lipid closer to the lipid/water interface where an interaction with the water could be seen by the hydroxyl group (-OH). Knowledge on localization in the formulation and dynamic processes such as aggregation that results in poor solubility can be obtained from the developed MD for lipid-based formulations. MD shows the patterns of different compounds in physical behaviour. It serves as the basis for in-silico design of lipid formulation [153,154,155,156,157,158].

## 11. Future Perspectives and Conclusions

The LBDDS have shown enormous potential for improving absorption and oral bioavailability of medicinal products that are poorly soluble in water. A proper understanding of drug physicochemical properties and nature of lipid excipients including design thinking such as quality by design approaches and in vivo performance is required before initiating the product development. The process responsible for enhancing the bioavailability of drugs is very different from other delivery systems, and these systems require the use of complex methods of characterization to understand the fate of GIT transit formulation. It is still a major challenge to establish robust and simple methods for the in vitro-in vivo correlation of LBDDS. Weak stability and difficult manipulation due to their liquid nature have been the major challenges that have limited LBDDS in the past. With different methods for the solidification of liquid formulations, these challenges have been effectively unravelled. The use of these industrial techniques, such as freeze-drying, spray-drying, spray-coating, and granulation on inert pellets, enabled the manufacture of these products on a large scale, triggering the expansion of their market availability. We here covered the mechanism of drug absorption, the role of MD in solubility and pharmacokinetics prediction, and regulatory perspectives in designing, developing lipidic products mostly for poorly aqueous soluble drugs. Guidelines and experimental methods need to be established for lipid formulations at early stages. During capsule filling, solution stability and capsule compatibility need to be understood, especially for drugs predisposed to oxidation and sensitive to moisture. Relevant strategies to examine in vitro and in vivo drug solubilization to predict the dynamic changes that occur in the gut after administration of the formulation are need to be explored.

## Figures and Tables

**Table 1:** List of lipid excipients used in the pharmaceutical product development USFDA IID- United States of Food and drug administration inactive ingredient database, USP—United States Pharmacopeia, NF—National formulary.

Chemical Name	Functionalities	Example	Regulatory Status
Caprylocaproyl macrogol-8 glycerides	Solubilizer	SMEDDS [8]	FDA IID
Hard Fat	Taste masking, Hot melt coating	HME, Melt granulation, NLCs [9]	USFDA
Glycerol dibehenate	Sustained-release, Lubricant, API protection	SR tablet, SLN and NLCs [10]	FDA IID
Glyceryl Monostearate	Lubricant, Emulsifier	Colloidal carriers [11]	USFDA
Glycerol distearate	Sustained release, Lubricant, Taste-masking	SR Tablet, Lipid nanoparticles, Hot melt coating [12]	FDA IID
Glyceryl Monocaprylate	Permeability enhancer, Emulsifier, Solubilizer	SEDDS [13]	USP/NF
Propylene Glycol Monocaprylate	Emulsifier, Solubilizer	SEDDS, Nanoparticles [13]	USFDA
Glycerol Monocaprylocaprate	Solubilizer, penetration enhancer	SNEDDS [14]	USFDA
Succinic Triglycerides	Super fatting Agent	Creams [15]	USFDA
Lauroyl macrogol-32 glycerides	Bioavailability enhancer	Hard gelatin capsule [15] (SNEDDS, SMEDDS), Mixed micelles [16]	FDA IID
Oleoyl macrogrol-32 glyceride	Bioavailability enhancer, Solubilizer	Oral solutions, SMEDDS [17]	FDA IID
Polyglyceryl-3 dioleate	Surfactant	Niosomes [18]	FDA IID
Propylene glycol dicaprylocaprate	Oily vehicle	Emulsions and Nanocapsules [19]	FDA IID
Trimyristin	Controlled release, Lubricant, taste masking	SLNs [20], HME, SEDDS, SLMs [20]	USFDA
Tristearin/Glyceryl Tristearate	Controlled release, Lubricant, lipid carrier	HME, Hot melt coating as a taste masking agent, SMEDDS and SLMs [20,21]	USFDA
Glyceryl Caprylate	Permeability enhancer, Emulsifier, Solubilizer	SMEDDS [22,23]	IIG/USFDA
Polyoxyl stearate	Bioavailability enhancer	(SNEDDS, SMEDDS) and Solid dispersion [23,24,25,26]	FDA IID
Stearoylmacrogol-32 glyceride	Bioavailability enhancer	Solid dispersion [26]	FDA IID

**Table 2:** Summary of application of lipid excipients.

S. No.	Drug	Formulation	Excipients	Application	References
1	Olanzapine	NLCs	Glyceryl monostearate, Tripalmitate, polyoxypropylenepolyoxyethylene block copolymer (pluronic F-68)	Enhancement in oral bioavailability bypassing pre systemic metabolism	Jawahar N. et al. [92]
2	Curcumin	Emulsion	Corn oil, polyethylene sorbitan monooleate	Improved bioavailability	Liqiang et al. [93]
3	Hydrochlorothiazide	SLN	Glyceryl distearate, polyoxypropylenepolyoxyethylene block copolymer (pluronic F-68) and polyethylene sorbitan monooleate	Increased diuretic effect and bioavailability	Cirri et al. [94]
4	Abiraterone acetate	Silica-lipid hybrids	Propylene glycol monocaprylate, glyceryl caprylate, PEG-35 castor oil	Exceeded the bioavailability by 1.43 fold	Schultz et al. [95]
5	Paclitaxel	Microemulsion	Distilled diacetylated monoglyceride, lecithin, glyceryl caprylate	p-gp efflux inhibition, increase in in situ permeability	Nornoo et al. [96]
6	Mupirocin	NLC	Cetyl palmitate, caprylic/caprylic acid, polyethylene sorbitan monooleate, phosphatidylcholine	preventing enzymatic degradation	Alcantar et al. [97]
7	Quercetin	Mixed micelles	Polyoxazolines, phosphatidylcholine	Improved antioxidant effect	Simon et al. [98]
8	Ibuprofen	Mixed micelles	Sodium deoxycholate, polyethylene sorbitan monooleate	Enhanced topical delivery	Sun et al. [4]
9	Chloraluminumphthalocyanine	Liposomes	egg phosphatidylcholine	Reduced parasitic load in liver and spleen	Lopes SC et al. [99]
10	Allantoin	Liposomes	Argan oil, phospholipids	Enhanced penetration	Manca et al. [100]
11	*trans*-resveratrol	Microemulsions	PEG-35 castor oil,1,2-propanediol, essential oils	Enhanced skin permeation	Cong et al. [101]
12	Methotrexate and etanercept	SLNs	Cetyl palmitate	improved drug therapeutic effect	Ferreira et al. [102]
13	Ferrous chlorophyllin	Ethosomes	Soya Lecithin, ceteareth-25	Increased skin penetration	Simone et al. [103]
14	Dexamethasone acetate	SLNs	Soybean lecithin, glycerol tristearate, pluronic F-68	Higher uptake by the lung	Xiang et al. [104]
15	Lipofectamine^®^	Lipoplexes	1,2-dioleoyloxy-3(trimethylammonium)propane, dioleoylphosphatidylethanolamin, DNase I	1.3-fold higher tumor transfection	Kumar et al. [105]

## Data Availability

Not Applicable.
